# Carbapenem-Resistant Enterobacteriaceae Infections: Taiwan Aspects

**DOI:** 10.3389/fmicb.2018.02888

**Published:** 2018-11-27

**Authors:** Shio-Shin Jean, Nan-Yao Lee, Hung-Jen Tang, Min-Chi Lu, Wen-Chien Ko, Po-Ren Hsueh

**Affiliations:** ^1^Department of Emergency, School of Medicine, College of Medicine, Taipei Medical University, Taipei, Taiwan; ^2^Department of Emergency Medicine and Department of Emergency and Critical Care Medicine, Wan Fang Hospital, Taipei Medical University, Taipei, Taiwan; ^3^Department of Internal Medicine, National Cheng Kung University Medical College and Hospital, Tainan, Taiwan; ^4^Department of Medicine, Chi Mei Medical Center, Tainan, Taiwan; ^5^Department of Health and Nutrition, Chia Nan University of Pharmacy and Science, Tainan, Taiwan; ^6^Department of Microbiology and Immunology, School of Medicine, China Medical University, Taichung, Taiwan; ^7^Department of Laboratory Medicine, National Taiwan University Hospital, National Taiwan University College of Medicine, Taipei, Taiwan; ^8^Department Internal Medicine, National Taiwan University Hospital, National Taiwan University College of Medicine, Taipei, Taiwan

**Keywords:** carbapenem-resistant Enterobacteriaceae, carbapenemase, *Klebsiella pneumoniae*, *Escherichia coli*, long-term care facility, tigecycline, colistin, avibactam

## Abstract

Carbapenem-resistant Enterobacteriaceae (CRE), a major resistance concern emerging during the last decade because of significantly compromising the efficacy of carbapenem agents, has currently become an important focus of infection control. Many investigations have shown a high association of CRE infections with high case-fatality rates. In Taiwan, a few surveys observed that a significant proportion (29–47%) of the CR-*Klebsiella pneumoniae* isolates harbored a plasmidic allele encoding *K. pneumoniae* carbapenemases (KPC, especially KPC-2). A significant increase in the number of oxacillinase (OXA)-48-like carbapenemases among CR-*K. pneumoniae* isolates was observed between 2012 and 2015. By striking contrast, isolates of CR-*Escherichia coli* and CR-*Enterobacter* species in Taiwan had a much lower percentage of carbapenemase production than CR-*K. pneumoniae* isolates. This differs from isolates found in China as well as in the India subcontinent. Apart from the hospital setting, CRE was also cultured from the inpatients from communities or long-term care facilities (LTCF). Therefore, implementation of regular CRE screening of LTCF residents, strict disinfectant use in nursing homes and hospital settings, and appropriate control of antibiotic prescriptions is suggested to alleviate the spread of clinical CRE isolates in Taiwan. Although there are some promising new antibiotics against CRE, such as ceftazidime-avibactam, meropenem-vaborbactam, aztreonam-avibactam and cefiderocol, these agents are not available in Taiwan currently. Therefore, in order to effectively decrease case-fatality rates among patients with the infections owing to carbapenemase-producing CRE isolates, combination antibiotic schemes, including colistin (or amikacin) and/or tigecycline in combination with an anti-pseudomonal carbapenem agent, remain the mainstay for treating clinical CRE infections.

## Introduction

Carbapenem-resistant Enterobacteriaceae (CRE), drawing great attention because of their serious resistance spectra and outbreak episodes in the northeastern United States since about two decades ago, have shown a high potential of rapid disseminations (Bratu et al., 2005a). Infections caused by CRE with production of various carbapenemases have become a major global worrisome concern due to their association with high (>30%) case-fatality rates in the current antibiotic pipeline (Tumbarello et al., 2012; Tzouvelekis et al., 2012; Navarro-San Francisco et al., 2013; Jean et al., 2015). In addition, McConville et al. conducted an observational study investigating CRE colonization (by rectal swabs) with subsequent impact on clinical outcomes for patients requiring admission to an intensive care unit (ICU) (McConville et al., 2017). By multivariate analysis, they found that CRE colonization also independently predicted development of a further systemic CRE infection at 30 days [adjusted odds ratio (aOR), 10.8; 95% confidence interval (CI), 2.8–41.9, *P* = 0.0006]. In 2017, the World Health Organization ranked CRE one of the first antibiotic-resistant “critical priority pathogens” (World Health Organization [WHO], 2017). Nevertheless, among these CRE isolates, the carbapenem non-susceptibility phenotypes are attributed to production of carbapenemase(s), or more likely, production of extended-spectrum β-lactamase (ESBL) plus AmpC β-lactamase with dysfunctional entry routes (i.e., porin change) of carbapenems, integrons and insertion sequence common region 1 (ISCR1) carrying various resistance genes, and/or efflux pumps, etc. (Doumith et al., 2009; Yang et al., 2012; Rui et al., 2018). To alleviate the resistance burden, close monitoring of CRE prevalence rates, as well as investigation of carbapenemase types, has become the main foci for infection control in most countries (Gupta et al., 2011).

Between 2004 and 2013, the prevalence rates of CRE among all clinical Enterobacteriaceae isolates have gradually risen (up to about 7%) in medical centers and major regional teaching hospitals throughout Taiwan. However, official surveillance data, conducted by Taiwan Centers for Disease Control (Taiwan CDC), clearly showed the annual prevalence rates of overall CRE isolates collected from ICUs rose from 3.7% in 2008 to 15.3% in 2017 (data until the third quarter) (Taiwan Nosocomial Infection Surveillance [TNIS], 2017). In addition, among *Escherichia coli* isolates acquired at ICUs, CRE rates rose from 1.2% in 2008 to 4.0% in 2017 at medical centers (bed number, >1000) (until the third quarter), while CRE rates in major regional teaching hospitals (bed number, 500–1000) rose from 1.0% in 2008 to 2.8% in 2017. These data might reflect the differences in CRE burden between hospitals of various care levels. By striking contrast, during the same period, significantly higher CRE rates for ICU-acquired *Klebsiella pneumoniae* isolates were observed in medical centers (from 6.1 to 28.2%) and in major regional teaching hospitals (from 3.7 to 24.8%) than those of ICU-acquired *E. coli* (Taiwan Nosocomial Infection Surveillance [TNIS], 2017). It was determined by multivariate logistic regression analyses that clinicians in Taiwan (aOR, 1.73; 95% CI, 1.03–2.92) and in the US (aOR, 1.89; 95% CI, 1.05–3.39) are more likely to prescribe carbapenem antibiotics to treat the potential ESBL-producing Enterobacteriaceae infections instead of β-lactam combination agents compared to other countries (Harris et al., 2017). Clinical overuse of carbapenem agents unavoidably result in increased drug resistance (Tumbarello et al., 2012; Sheu et al., 2018).

## Study Designs

We searched and reviewed literature from the 2002–2018 PubMed database containing important keywords, including “carbapenem-resistant Enterobacteriaceae,” “carbapenemase-producing Enterobacteriaceae,” “prevalence rates,” “mortality,” “case-fatality,” “Taiwan,” “Centers for Disease Control,” “*Klebsiella pneumoniae*,” “*Klebsiella pneumoniae* carbapenemase (KPC),” “New Delhi metallo-β-lactamase (NDM),” “oxacillinase-48 (OXA-48)-like,” “*Escherichia coli*,” “*Enterobacter* species,” “resistance mechanisms,” “community-acquired,” “long-term care facility,” “Clinical and Laboratory Standards Institute,” “European Committee on Antimicrobial Susceptibility Testing,” “monotherapy,” “combination therapy,” “tigecycline,” “colistin,” and “novel antibiotics.”

## Prevalence and Mortality Rates of Carbapenemase-Producing Enterobacteriaceae (CPE) Among CRE

In Pakistan, spread of special sequence types (STs; ST15 and ST48) of multidrug-resistant (MDR) *Klebsiella* species (most were ESBL-producing *K. quasipneumoniae*) isolates was observed since 2010 at one tertiary-care hospital (Ejaz et al., 2017). Similarly, among 83 carbapenem-resistant *K. pneumoniae* isolates studied in Brazil, a clinical isolate of KPC-2 and OKP-B-6 β-lactamase-producing *K. quasipneumoniae* subsp. *similipneumoniae* was also reported. (Nicolás et al., 2018). By stark contrast, global dissemination of CPE (especially *K. pneumoniae* isolates) in fact has been occurring at an alarming pace for many years (Logan and Weinstein, 2017). In Taiwan, most CP-*K. pneumoniae* isolates are the KPC producers (Jean et al., 2015). The *bla*_KPC_ genes mostly reside on transferable plasmids which contain transposase, resolvase, and mobile transposons (Tn*4401*), thereby posing a formidable threat to infection control (Nordmann et al., 2009). As compared to other Enterobacteriaceae species, it is noteworthy that these mobile transposons are only detected among few epidemic clones of KPC producers of *K. pneumoniae* with distinct STs (predominantly ST258 in US and Israel, while ST11 in China and Taiwan) (Naas et al., 2008). The ST11 clone was the most prevalent ESBL-producing clone (particularly CTX-M-15, CTX-M-14, and SHV-5 types) of *K. pneumoniae* isolates in many Asian countries (Lee et al., 2011; Ma et al., 2013a).

Regarding the molecular analyses of carbapenemase types in CRE in Taiwan, a multicenter study first examined imipenem-resistant *K. pneumoniae* isolates collected from 13 Taiwanese hospitals from 2002 through 2009 (Ma et al., 2013a). This study revealed that a majority (84.6%) of *K. pneumoniae* strains (mainly ST11 clone) with > 2 mg/L minimum inhibitory concentrations (MICs) to imipenem harbored *bla*_IMP-8_, plus various genes encoding ESBLs in combination with loss of porins (mainly OmpK35) (Ma et al., 2013a). In 2012, a small-scale Taiwanese study, conducted by Chang et al., investigated 66 patients with CRE (comprising *K. pneumoniae* and *E. coli*) infections (*n* = 46) or colonization (*n* = 20). It showed that the CPE prevalence rate was 28.8% (*n* = 19; 14 of which harbored *bla*_KPC-2_), and a 30-day mortality rate was 50% (23/46) among patients with CRE infections (Chang et al., 2015). The CPE prevalence of Chang’s study was different from that [11.5%; most were metallo-β-lactamase (MβL) producers] of the study conducted by Javed et al. (2016) in Pakistan during 2013–2014. In addition, this mortality rate was higher compared to that (40.8%) of the other CRE study (susceptibility evaluated by the disk diffusion method, and data interpretation in accordance with the 2009 criteria) at a medical center located in the middle-western part of Taiwan during 2010–2011 (Huang et al., 2014). In Chang’s study, nearly one-half [47.8% (22/46)] of the infections originated in the lower respiratory tract. Furthermore, patients with a co-morbidity of diabetes mellitus, initially presenting with shock or high scores of the Acute Physiology and Chronic Health Evaluation (>23 points), or receiving non-susceptible antibiotic regimen therapy (regardless of single or combination drug therapy) for > 48 h had significantly higher case-fatality rates (*P* < 0.05) than the other factors by the univariate analysis (Chang et al., 2015). Of note, patients who acquired CRE infections owing to KPC (dominant carbapenemase type)-producing Enterobacteriaceae also had a trend toward more fatal outcomes than those without KPC (*P* = 0.14) (Chang et al., 2015). An additional CRE study conducted at a single medical center of northern Taiwan during 2012–2013 showed that a CRE isolate with an imipenem MIC ≥ 16 mg/L independently predicted 14-day mortality among patients regardless if the isolate was from infection or colonization (Wu et al., 2016). In the survey published by Wu et al., KPC-2 was the dominant (87.1%) carbapenemase among CPE (29.5% of overall CRE), and the in-hospital mortality rate among patients with CR-*K. pneumoniae* was 43.8% (Wu et al., 2016). Wang et al. examined 1135 clinical isolates of various Enterobacteriaceae species collected from four major hospitals in Taiwan between 2010 and 2012. Fifty-seven isolates were carbapenemase-producing Enterobacteriaceae (CPE) isolates (5%), 54.4% of the CPE isolates co-harbored *bla*_ESBL_ alleles. Furthermore, Wang et al. found that *Enterobacter cloacae* isolates predominantly harbored the *bla*_IMP-8_ allele (26/27), while isolates of CP-*K. pneumoniae* mainly harbored *bla*_KPC-2_ alleles (16/17; ST11 clone predominated) (Wang et al., 2015). In addition, among Taiwanese CRE isolates surveyed, approximately one-half (45.8%) harbored various *bla*_ESBL_ genes (80% were the *bla*_CTX-M_ types), while 62.0% harbored alleles encoding various plasmid-mediated AmpC β-lactamases (70.6% were *bla*_DHA_ and 22.3% were *bla*_CMY_) (Wang et al., 2015). The high rates of *K. pneumoniae* isolates co-harboring ESBL and/or AmpC-encoding alleles among CRE corresponded well with those of an Asia-Pacific study with respect to Enterobacteriaceae isolates responsible for complicated intra-abdominal infections (cIAI) and complicated urinary tract infections (cUTI) from 2008 through 2014 (Jean et al., 2017). According to the above findings, instead of carbapenemase production, ESBL with concomitant AmpC production and membrane impermeability caused by porin loss obviously more likely confers *in vitro* non-susceptibility to carbapenem agents (especially ertapenem) among CRE in Taiwan and the Asia-Pacific region. These results are similar to that found in Hong Kong (Ho et al., 2016).

**FIGURE 1:**
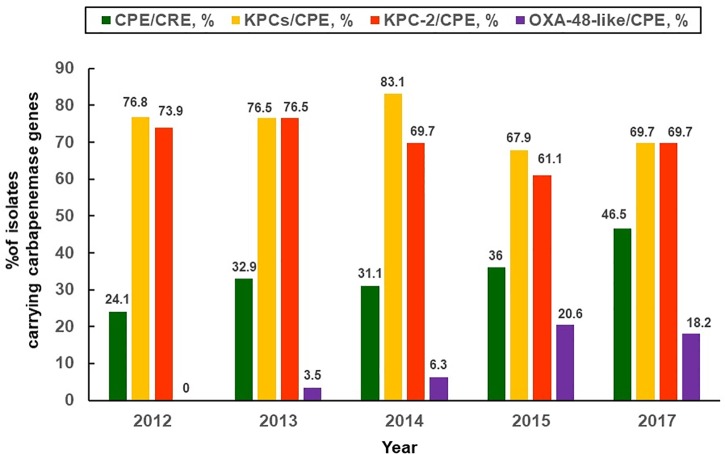
Distribution of carbapenemases. Annual proportions of overall and different types of carbapenemase producers (*Klebsiella pneumoniae* carbapenemase, oxacillinsase-48-like) among carbapenem-resistant *K. pneumoniae* isolates collected between 2012 and 2017 in Taiwan (Chiu et al., 2018; Jean et al., 2018b).

Despite non-CPE isolates account for a majority of CRE in Taiwan, an up-surging CPE trend [three-fourths (74.5%) of CPE harboring *bla*_KPC-2_ and were ST11 clone, followed by CPE harboring *bla*_V IM-1_ allele (12.7%), etc.] was observed by Chiu et al. (2013). The CPE rates have increased from 0% in 2010 to 22.3% in 2012 among Taiwanese CR-*K. pneumoniae* isolates (showing > 1 mg/L of MICs to imipenem or meropenem) (Chiu et al., 2013). Furthermore, it is noteworthy that the prevalence rates of CPE [36.4%; mostly related to various *bla*_KPC_ (75.9%), followed by *bla*_OXA-48_-like (8.8%), *bla*_V IM-1_ (7.9%), and *bla*_IMP-8_ (5.7%), etc] among the studied CR-*K. pneumoniae* isolates (approximately 99% were non-susceptible to imipenem, and 85.8% were collected from medical centers) were estimated to have 1.5-fold increase from 2012 through 2015. Most significantly, an increasing trend was observed in the middle-western part of Taiwan (Chiu et al., 2018). Data regarding important CRE studies in Taiwan and other countries are shown in Table 1. There were big variations in the rates of CPE among clinical CRE isolates in Taiwan as compared to those from other countries (Lorenzoni et al., 2017; Woodford et al., 2017; Laolerd et al., 2018). It is notable that persistently high KPC-2 rates (73.9% in 2012, and 61.1% in 2015) and emergence of KPC-17 as well as KPC-34 since 2014 were observed (Chiu et al., 2018). As the *K. pneumoniae* of ST11 clone accounted for 86.9% of overall CP-*K. pneumoniae* isolates (Chiu et al., 2018), heightened infection control maneuvers are absolutely warranted for Taiwanese medical centers. Figure 1 illustrates the annual percentages of overall carbapenemase producers, overall KPCs and KPC-2 producers, as well as OXA-48-like producers among clinical CR-*K. pneumoniae* isolates (mainly non-susceptible to imipenem and ertapenem) collected between 2012 and 2015 in Taiwan (Chiu et al., 2018; Jean et al., 2018b).

**Table 1:** Studies of carbapenem-resistant Enterobacteriaceae in Taiwan and other countries.

Study periods	CRE case numbers, or CRE isolates under survey	Carbapenemase production, %	Outcomes	Reference
2010–2012	1,135 carbapenem non-susceptible Enterobacteriaceae isolates (from various clinical sources)	5% (57/1135)	NA	Wang et al., 2015
2012	66 (including 46 patients with diverse CRE infections and 20 patients with CRE colonization)	28.8% (19/66)	In-hospital mortality, 50% (23/46) for patients with CRE infections	Chang et al., 2015
2012–2013	105 (including 49 patients with various CRE infections, and 56 patients with CRE colonization)	29.5% (31/105)	Overall in-hospital mortality, 43.8% (46/105); for 49 cases with CRE infections, 63.3% (31/49)	Wu et al., 2016
2012–2015	1,457 CR-*K. pneumoniae* isolates	31.4% (457/1457)	NA	Chiu et al., 2018
2017	85 bloodstream CRE isolates, comprising *Escherichia coli* (*n* = 14) as well as *K. pneumoniae* (*n* = 71)	41.2% (35/85); 46.5% (33/71) for CR-*K. pneumoniae* isolates	NA	Jean et al., 2018b
2015	70 CRE isolates (from various clinical sources), collected from patients in Brazil	80% (56/70); all harbored *bla*_KPC_	NA	Lorenzoni et al., 2017
2016–2017	22 CRE isolates, collected from urine samples of patients in the United Kingdom	45.4% (10/22)	NA	Woodford et al., 2017
2017	287 clinical CRE isolates, collected from patients in Thailand	77.7% (223/287), a majority of them harbored *bla*_NDM-1_	NA	Laolerd et al., 2018

*CRE, carbapenem-resistant Enterobacteriaceae. NA, non-applicable*.

Carbapenem-resistant Enterobacteriaceae that only harbor *bla*_OXA-48_-like allele theoretically exhibit non-susceptibility to most carbapenem agents while sparing most β-lactam agents. However, most of the *bla*_OXA-48_-like-harboring CRE (especially *K. pneumoniae*) isolates are actually also the ESBL and/or AmpC co-producers (Saïdani et al., 2012; Girlich et al., 2014). Consequently, many OXA-48-like-producing CRE strains show *in vitro* extensively drug-resistant phenotypes. In 2014, an OXA-48-like carbapenemase-producing *E. coli* strain, which concomitantly harbored *bla*_CTX-M-1_, was cultured from pus sample of a Cambodian patient who resided in Taiwan (Jao et al., 2017). An additional 2010 study from India regarding CRE, some of which were CPE harboring *bla*_NDM_, *bla*_V IM_ or *bla*_OXA-181_-like alleles, revealed that the case-fatality rate was 56.7% (Mariappan et al., 2017). A sharp rise in the annual prevalence rate of OXA-48-like production among Taiwanese CPE isolates between 2012 and 2015 was also observed (Chiu et al., 2018) (Figure 1). Spread of *bla*_NDM_ alleles occurred rapidly in the Indian subcontinent during the last decade (Kumarasamy et al., 2010), and also in southeastern Asia (Vietnam and the Philippines) (Jean et al., 2017). Nevertheless, reports regarding the NDM-producing Enterobacteriaceae colonization in one patient (3 *K. pneumoniae* isolates harboring *bla*_NDM-1_, probably acquired from India) (Wu et al., 2010) and infection in the other patient (1 *Klebsiella oxytoca* isolate harboring *bla*_NDM-1_, acquired from China) (Lai et al., 2011) in Taiwan were rare (Jean et al., 2017).

## Community and Long-Term Care Facility (LTCF)-Acquired CRE

Few studies have addressed the percentage of community acquisition of clinical CRE infections in the PubMed database. However, a few investigations in Taiwan have addressed this important topic. A clinical study, conducted at a medical center located in southern Taiwan in 2015, showed approximately 30% (23/78) of cases, with predominance of elderly female patients and infections originating in the urologic system, acquired CRE in a community setting (Tang et al., 2016). The percentage of community-acquired CRE was higher than that [12% (14/117)] of another Taiwanese study conducted at a large medical center in 2010 (*P* = 0.002) (Lai et al., 2013), and also higher than that [5.6% (17/305)] of an US-CRE study (Thaden et al., 2014). In the latter study, 91% of overall CRE isolates were *K. pneumoniae*, while 59% were cultured from urine between 2008 and 2012 (*P* < 0.0001) (Thaden et al., 2014). In addition, it was observed that many residents from the LTCF in Taiwan were also the carriers of CRE, most (24/27) were *K. pneumoniae* (Chuang, 2015; Mao et al., 2018). This was similar to the study conducted by Lee C.M. et al. (2017) who observed that a significant proportion of *K. pneumoniae* strains cultured from rectal swabs of LTCF residents were CRE (38.9 and 88.9% showed *in vitro* non-susceptibility to ertapenem and imipenem, respectively). Therefore, in Taiwan, regular screening for CRE carriage (urine, sputum, and rectal swabs) among the LTCF residents to implement subsequent contact isolation precaution, hand hygiene, strict implementation of environmental disinfection (including equipment of LTCF), and adequate education to caregivers and family about the importance of infection control are beneficial to decrease CRE carriage rates of LTCF residents. All of these measures are crucial for preventing CRE spread at LTCF (Lee C.M. et al. (2017); Lai et al., 2018; Liu et al., 2018).

## Surveillance Data of Antimicrobial Resistance in Taiwan in 2017

To fully explore the evolutionary trends of the annual resistance burden, the Taiwan CDC conducted a consecutive 4-year survey of antimicrobial resistance (AMR) regarding important clinical bacterial pathogens since 2017 (Jean et al., 2018b). Among 673 bloodstream *K. pneumoniae* isolates collected from 16 major teaching hospitals throughout Taiwan in 2017, 71 (10.5%) displayed non-susceptibility to at least one carbapenem agent. In addition, a total of 92 (13.8%) *K. pneumoniae* isolates exhibited ESBL phenotypes that were unrelated to KPC and/or NDM production [with a 92% non-susceptible (NS) rate to piperacillin-tazobactam, 100% to ceftazidime, and 74% to cefepime]. Phenotypic ESBL isolates had high NS rates to ertapenem (40.2%), tigecycline [7.6% based on the US Food and Drug Administration (FDA) criteria, and 16.3% based on the criteria recommended by the European Committee on Antimicrobial Susceptibility Testing (EUCAST) in 2018 (European Committee on Antimicrobial Susceptibility Testing [EUCAST], 2018)], amikacin (13.0%), as well as ceftolozane-tazobactam [70.7% based on criteria recommended by Clinical, and Laboratory Standards Institute [CLSI], 2018, and 80.4% based on the EUCAST 2018 criteria]. Of note, among *K. pneumoniae* blood isolates validated as CPE by multiplex PCR tests, 24 (3.6%) harbored *bla*_KPC_ allele (1 isolate showed susceptibility to all tested carbapenem agents), eight (33.3%) of the KPC-producing *K. pneumoniae* isolates were acquired from a community setting. In addition, 7 (1.0%) *K. pneumoniae* isolates harbored *bla*_OXA-48_-like alleles (1 isolate showed susceptibility to all tested carbapenem agents, while 6 exhibited ESBL phenotypes), 1 (0.15%) and 4 (0.6%) isolates harbored *bla*_NDM_ and *mcr*-1 gene, respectively. The rate of KPC-producing isolates [32.4% (23/71)] among Taiwanese CR-*K. pneumoniae* isolates in 2017 was significantly higher than that (2.8%) in the 2010-2012 Taiwanese study (Wang et al., 2015). Furthermore, out of the total KPC-producing *K. pneumoniae* isolates (*n* = 24), there were two (8.3%) showing non-susceptibility to ceftazidime-avibactam, 5 (20.8%) showing non-susceptibility to colistin, and 4 (16.7%) displaying non-susceptibility to tigecycline based on the EUCAST 2018 criteria (Jean et al., 2018b, in Infect Drug Resist).

## Carbapenem Resistance Mechanisms Among Cr-*E. coli* Isolates

As compared to a much higher CPE rate among clinical isolates of *K. pneumoniae*, two Taiwanese studies concluded that co-existence of a plasmidic AmpC β-lactamase (DHA-1, CMY-2) in combination with loss of an outer membrane porin (OmpC/F) is the main mechanism responsible for non-susceptibility to carbapenems for CR-*E. coli* (Chia et al., 2009; Ma et al., 2013b). This finding differs from that in Mainland China [89% of CR-*E. coli* isolates (*n* = 164) harbored *bla*_NDM_ (*n* = 81) or *bla*_KPC_ (*n* = 65) alleles] (Zhang et al., 2017), and in India (most CR-*E. coli* harbored *bla*_NDM_ with or without *bla*_OXA-48_-like alleles) from 2011 through 2013 (Mohanty et al., 2017). Surveillance data from the Taiwan CDC-AMR 2017 showed a total of 686 *E. coli* blood isolates were collected. Among them, only 14 (2.0%) isolates showed *in vitro* non-susceptibility to ertapenem. Of note, 5 (0.7%) isolates harbored the *mcr*-1 gene, similar to the one found in Mainland China (Quan et al., 2017), while the other two harbored a *bla*_KPC_ and *bla*_NDM-1_ allele, respectively.

## Carbapenem Resistance Mechanisms Among Cr-*Enterobacter* spp. Isolates

Fewer studies have examined mechanisms of non-susceptibility to carbapenem agents among isolates of *Enterobacter* species than those for *K. pneumoniae* in the PubMed database. A study focusing on ertapenem-resistant *Enterobacter cloacae* isolates (MIC > 2 mg/L, in accordance with the CLSI 2009 criteria) collected in 2007 was conducted by Yang et al. (2012) in Taiwan. Analysis of porin expression, detection of efflux pumps and β-lactamase(s), as well as susceptibility tests were performed. Only a few isolates [3/53 (5.7%)] harbored *bla*_IMP-8_, while porin change (30–40%) and efflux pump(s) (≥70%) in combination with ESBL or AmpC significantly contributed to ertapenem non-susceptibility for Taiwanese ertapenem-resistant *E. cloacae* isolates. Because of geographic variations, production of different types of carbapenemases among the carbapenem (imipenem, ertapenem)-NS *E. cloacae* isolates was observed in India (VIM-2, VIM-6, and NDM-1) (Khajuria et al., 2014) and in Israel (KPC-2) (Marchaim et al., 2008).

## Monotherapy Vs. Combination Therapy for CRE Infection

Patients with CPE infections are undoubtedly at an extremely high risk for inappropriate antibiotic therapy (Tumbarello et al., 2012; Chang et al., 2015). As stated earlier, tigecycline and colistin showed good *in vitro* susceptibility results against most CRE isolates and KPC or MβL-producing CPE isolates (Bratu et al., 2005b; Castanheira et al., 2008). As stated in the Taiwan CDC-AMR 2017 data, the tigecycline/colistin MIC values of one *E. coli* and one *K. pneumoniae* isolate harboring the *bla*_NDM_ allele were 0.06/0.25 mg/L and 0.25/0.25 mg/L, respectively. Nevertheless, to maximize the clinical effectiveness of colistin against impending resistant Gram-negative bacteria (GNB; i.e., MIC of colistin is 2 mg/L), a study showed that both the loading, as well as maintenance dose should be increased (Garonzik et al., 2011). In addition, tigecycline was validated as a bacteriostatic agent with suboptimal concentrations in bloodstream (<2 mg/L) after administration of the standard-dose regimen (Yahav et al., 2011). Consequently, monotherapy with tigecycline or colistin is not recommended in the treatment of severe GNB infections owing to high clinical failure and superinfection rates. Bass et al. found that combination therapy of multiple agents that have appropriate *in vitro* activities for ≥ 48 h was associated with improved survival rates (OR, 0.19; 95% CI, 0.06 to 0.56; *P* < 0.01) for critically ill patients with CR-GNB bacteremia (regardless of Enterobacteriaceae species or non-fermentative GNB) (Bass et al., 2015; Lee C.H et al., 2017). Many combination therapies were also been shown to result in favorable outcomes in patients with CP-GNB. For example, a combination of doripenem with colistin was reported to have an excellent *in vitro* synergistic effect against CP-*K. pneumoniae* isolates (Jernigan et al., 2012; Rodríguez-Baño et al., 2018). In addition, a tigecycline-colistin combination was also shown to be superior to colistin monotherapy in decreasing future resistance to colistin (Lee et al., 2009).

In Taiwan, data comparing therapeutic efficacy between different antibiotic regimens against CRE or CPE were scarce (Lee et al., 2011; Ku et al., 2017). Although the rate of tigecycline non-susceptibility among Taiwanese CRE (*E. coli* plus *K. pneumoniae*) blood isolates in 2017 was not high [3.5% (3/85) based on FDA criteria and 12.9% (11/85) based on the EUCAST 2018 criteria], a Taiwanese study aiming specifically at 16 tigecycline-resistant *K. pneumoniae* strains elucidated that *ramR* deficiency and/or widespread mutated *tet*(A) are the main mechanisms conferring non-susceptibility to the tetracycline-class agents for these *K. pneumoniae* isolates (Chiu et al., 2017). In addition, acquisition of fosfomycin-resistant genes, *fosA* subtypes and *fosC2*, which were mainly transmitted by plasmids and/or transposons, was found in many of the Enterobacteriaceae species in Eastern Asian countries, including Taiwan (Yang et al., 2017). In addition, although amikacin, gentamicin and fosfomycin exhibited acceptable *in vitro* susceptibility rates against some CPE species (KPC and VIM) (Endimiani et al., 2010; Lorenzoni et al., 2017), these agents are recommended as mere adjunctive options against critical CP-GNB infections because of rapid induction of resistance after monotherapy (Tseng et al., 2017; Yang et al., 2017). By contrast, against the non-CP-CR-*K. pneumoniae* strains (with changes of out-membrane porins) collected from a medical center of southern Taiwan during 2008–2012, Ku et al. used the sub-inhibitory concentrations (1/2 × MIC) of antibiotics under evaluation and time-kill studies to analyze *in vitro* synergism between different combination regimens. As compared to tigecycline plus fosfomycin and colistin plus fosfomycin, Ku et al. observed that tigecycline in combination with colistin showed much better *in vitro* synergism, as well as bactericidal efficacy (Ku et al., 2017).

## Clinical Evidence of Combined Antimicrobial Schemes Against CPE Infections

Although the above *in vitro* synergy data supported the use of combination regimens, well-designed clinical randomized control trials that strictly investigated which antimicrobial schemes are effective against KPC-producing Enterobacteriaceae isolates were few. Nevertheless, there were many retrospective studies in favor of the use of various combination therapy regimens against clinical CPE infections. None of these studies were investigated in Taiwan. The recommended antibiotic regimens included anti-pseudomonal carbapenem plus colistin or tigecycline, polymyxin plus tigecycline or aminoglycoside, or meropenem in combination with tigecycline and colistin. Important data regarding therapeutic efficacy of different antibiotic regimens (various combination schemes vs. monotherapy) for patients with CPE infections are illustrated in Table 2 (Lee and Burgess, 2012; Qureshi et al., 2012; Tumbarello et al., 2012; Tzouvelekis et al., 2012, 2014; Daikos et al., 2014; Food and Drug Administration [FDA], 2018; Rodríguez-Baño et al., 2018).

**Table 2:** Clinical efficacy of different antibiotic regimens on patients with clinical infections due to isolates of carbapenemase-producing Enterobacteriaceae.

Study periods	Carbapenemase production	Antibiotic regimens	Clinical outcomes	Reference
2005–2009	41 patients, with KPC-producing *K. pneumoniae* bloodstream infections	Carbapenem + colistin, or tigecycline	28-day mortality rates, 13% for combination group vs. 67% for colistin or tigecycline monotherapy	Qureshi et al., 2012
2001–2011 (review of MEDLINE database)	Diverse infections, due to isolates of KPC-producing *K. pneumoniae*	Polymyxin + carbapenem, tigecycline, or aminoglycoside	Treatment failure rates, 25% vs. 49% for combination therapy vs. monotherapy group (73% in polymyxin, and 60% in carbapenem monotherapy group)	Lee and Burgess, 2012
2010–2011	125 patients, with KPC-*K. pneumoniae* bloodstream infections	Meropenem + tigecycline + colistin (regimen of (triple combination)	Mortality rates, 13% for triple combination therapy vs. 42% monotherapy group	Tumbarello et al., 2012
2001–2010	Patients with CPE septicemia, including primarily bacteremia (*n* = 244), and pneumonia (*n* = 32), etc. Among CPE isolates, 158 KPC + 140 MβL producers predominantly	Carbapenem + colistin/or aminoglycoside	Mortality rates, 6% vs. > 23% for >2 *in vitro* active drugs vs. non-susceptible drugs	Tzouvelekis et al., 2012
2009–2010	205 patients, with bloodstream CPE (*K. pneumoniae*) infections due to 163 KPC + VIM producers, and 42 VIM producers	Two *in vitro* active drugs	28-day mortality rates, 27.2% (with MICs of *K. pneumoniae* to imipenem, meropenem, or doripenem < 8 mg/L) plus a second *in vitro* active agent vs. 44.4% for monotherapy group	Daikos et al., 2014
2007–2014 (data were pooled from 20 clinical studies)	414 patients, with diverse infections due to CPE, mainly harboring KPC or VIM alleles	Heterogeneous regimens, including the carbapenem-containing vs. carbapenem-sparing schemes	Mortality rates, 18.8% for carbapenem-containing therapy group vs. 30.7% for carbapenem-sparing therapy group.	Tzouvelekis et al., 2014

*KPC, Klebsiella pneumoniae carbapenemase. CPE, carbapenemase-producing Enterobacteriaceae. MβL, metallo-β-lactamase. VIM, Verona integron-encoded metallo-β-lactamases*.

## Other Antimicrobial Agents Against CPE Infections

Apart from tigecycline and colistin against CPE, previous studies showed that avibactam, a bridged diazabicyclo octanone, exhibited good *in vitro* activity against GNB with serine β-lactamases (especially against the KPC producers) when combined with ceftazidime, but it is inactive against class B β-lactamases (Bonnefoy et al., 2004; Tzouvelekis et al., 2012; van Duin and Bonomo, 2016). In the Taiwan CDC-AMR 2017 survey, the MICs for ceftazidime-avibactam for one *E. coli* and one *K. pneumoniae* isolate that harbored the *bla*_NDM_ allele were both > 64 mg/L. Consequently, the Taiwanese 2017 data were consistent with previous studies. Ceftazidime-avibactam has been approved by the US-FDA in treatment of CRE-related cIAI and cUTI. In addition, meropenem-vaborbactam has an equivalent potency to ceftazidime-avibactam against class A carbapenemases (typified by KPC), but also has limited activity against class B along with oxacillinase carbapenemases (Castanheira et al., 2017; Chew et al., 2018). This drug was also approved in treatment of CRE-related cUTI recently. By contrast, aztreonam-avibactam displays good *in vitro* susceptibility in dual-carbapenemase (KPC, NDM)-producing CRE isolates (Chew et al., 2018). In addition, cefiderocol, a new siderophore cephalosporin, also shows potentially good *in vitro* activity against both KPC, as well as NDM-producing Enterobacteriaceae isolates (Hackel et al., 2018). The clinical trials comparing clinical efficacy of aztreonam-avibactam and cefiderocol with other best available antibiotics are undergone. The other future promising agents against CPE include imipenem-relebactam, plazomicin, and eravacycline (Rodríguez-Baño et al., 2018).

## Antibiotic Treatment for Non-Carbapenemase-Producing-CRE Infections

Compared to ample databases regarding treatment of CPE, limited data were available regarding treatment recommendations for clinical non-CP-CRE isolates. Nevertheless, Tamma et al. observed MIC values for imipenem and meropenem in the subgroup of CPE isolates (>90% were KPC producers) were significantly higher than those of the non-CP-CRE subgroup. Furthermore, the overall 14-day mortality rate was 4-fold higher among patients with CPE bacteremia than those of the non-CP-CRE group (Tamma et al., 2017). Recently, we investigated detection of CPE predictors among ertapenem-NS Enterobacteriaceae isolates causing IAI collected from patients hospitalized in Asia-Pacific countries during 2008–2014 (Jean et al.,2017). This Asia-Pacific IAI-CRE study showed that imipenem non-susceptibility (i.e., MIC > 2 mg/L) and culturing ertapenem-NS Enterobacteriaceae isolates from the peritoneal space were highly likely as CPE (dominated by the *bla*_NDM_ and *bla*_IMP_-harboring isolates, rather than KPC producers; Jean et al., 2018a, in Infect Drug Resist). Moreover, the non-CP-ertapenem-NS IAI Enterobacteriaceae isolates were more likely to have cefepime MICs of > 8 mg/L, which might be valuable in distinguishing it from the CPE. However, the clinical application of the dose adjustment of an anti-pseudomonal carbapenem agent to effectively treat the non-CP ertapenem-NS Enterobacteriaceae isolates needs further investigation in clinical scenarios.

## Summary

In Taiwan, the main research gap is that there are no sufficient clinical studies exploring the significant risk factors with respect to acquisition of CRE isolates among hospitalized or LTCF patients. Of note, the annual CPE proportion has been on a sharp rise among clinical CRE isolates since 2012, particularly for CR-*K. pneumoniae* isolates. To effectively limit the spread of CPE (especially ST11 *K. pneumoniae* clone) in clinical settings, close monitoring of this worrisome MDR trend is warranted at all major teaching hospitals, as well as nursing homes. In addition, although ceftazidime-avibactam and meropenem-vaborbactam show excellent efficacy against some CPE, it is not currently available in Taiwan. Combination therapy schemes, such as colistin and/or tigecycline plus an anti-pseudomonal carbapenem agent, are still the preferred treatment for CRE and CPE infections in Taiwan.

## Author Contributions

S-SJ, N-YL, and P-RH collected and analyzed the data. S-SJ participated in the writing of the manuscript. S-SJ, N-YL, H-JT, M-CL, W-CK, and P-RH read and approved the final version of the manuscript.

## Conflict of Interest Statement

The authors declare that the research was conducted in the absence of any commercial or financial relationships that could be construed as a potential conflict of interest.
